# Insanitary Areas and Rehousing

**Published:** 1898-12-24

**Authors:** 


### INSANITARY AREAS AND REHOUSING.

The resolutions of the London County Council of November 29th, according to which all clearances under the Housing of the Working Classes Act, 1890, which involve rehousing are to be done at the sole cost of the Council, has not been long in bearing fruit. The more impecunious vestries are already looking out for areas to be cleared, and for sites to be built upon, at the expense of the ratepayers at large, and among them the parish of St. George-the-Martyr, with its energetic medical officer, Dr. Waldo, is well to the fore. Already a special report on unhealthy areas and on vacant sites has been drawn up, and the vestry is being urged, without loss of time, to make representations to the County Council on the subject. We do not, however, gather that according to the resolutions the Council is in any way bound to rehouse in the same parish as that in which the clearance is made, and it is much to be hoped that, as the Council is now committed to building, it will take care to obtain sites for the purpose on something like a general plan, and one which shall lessen instead of merely perpetuating the present overcrowding. Of course, each vestry would like to have money from the general purse spent within its own boundaries; but when Dr. Waldo shows, as he does in his report, that the population density of St. George's is at the rate of 212 persons per acre, and when he uses that fact as a proof of overcrowding, and as showing that the Council should intervene, he must allow us to say that the argument cuts both ways, and that this same fact can be urged with equal force against the spending of public money for the perpetuation of the overcrowding against which he protests. We hope that before the Council spends a penny on new sites for workmen's dwellings it will draw up a general plan of action, so that the excess of population shall be removed from the central parishes and located where the people can lead more healthy lives. We doubt, however, whether this is exactly what will please the parishes in question.

